# The impact of tuberculosis-induced hyperglycemia on pulmonary microbiota and airway mucus secretion in individuals not previously diabetic: A systematic review and meta-analysis protocol

**DOI:** 10.1371/journal.pone.0316810

**Published:** 2025-01-13

**Authors:** Victor Moses Musyoki, Marianne Mureithi, Annamari Heikinheimo, Elizabeth Maleche-Obimbo, Kariuki Njaanake, Omu Anzala

**Affiliations:** 1 Department of Medical Microbiology and Immunology, University of Nairobi, Nairobi, Kenya; 2 KAVI-Institute of Clinical Research, University of Nairobi, Nairobi, Kenya; 3 Tuberculosis and HIV Co-Infection Training Program, Kenya; 4 Department of Veterinary Medicine, University of Helsinki, Helsinki, Finland; 5 Department of Paediatrics and Child Health, University of Nairobi, Nairobi, Kenya; Endocrinology and Metabolism Population Sciences Institute, Tehran University of Medical Sciences, ISLAMIC REPUBLIC OF IRAN

## Abstract

The lung environment harbours a community of microbes that play a significant role in health and disease, including innate protection against pathogenic microorganisms. Infection with *Mycobacterium tuberculosis*, psychological stress associated with the tuberculosis (TB) disease, and the metabolites from the rifampicin treatment regimen have been reported to induce hyperglycemia and consequently type 2 diabetes mellitus (T2DM) in individuals not previously diabetic. The high glucose concentration is proposed to alter the composition of the lung microbiota and airway homeostasis, exerting an influence on TB disease and treatment outcomes. In this systematic review, we propose to synthesize literature on TB-induced hyperglycemia and its impact on lung microbiota and secretion of airway mucus in individuals not previously diabetic. A systematic search will be carried out on PubMed, EMBASE, MEDLINE, PROQUEST, Cochrane, SCOPUS, and manually on Google Scholar and references of relevant articles to identify other studies. We will review published articles that include studies on TB-induced hyperglycemia, pulmonary microbiome, mucin secretion, and (or) airway surface liquid upon TB diagnosis and during treatment. The quality of the study articles will be assessed using the modified Newcastle-Ottawa Scale (NOS). Meta-analysis will be conducted using random effect model for heterogeneity to pool estimates on microbial diversity. Egger’s test will be performed to explore any selective reporting bias. The findings of the systematic review and the meta-analysis will be reported as per the Preferred Reporting Items for Systematic Review and Meta-Analysis (PRISMA) protocol guidelines. This protocol was developed and uploaded onto the International Prospective Register of Systematic Reviews (PROSPERO) database, registration number: CRD42024482248.

## Introduction

Tuberculosis (TB) remains a global public health concern associated with significant morbidity and mortality [1]. In 2022, an estimated 7.5 million people were newly diagnosed with TB across the globe. This was the highest number of cases since World Health Organization (WHO) TB monitoring started in 1995; up from 5.8 million cases reported in 2020 and 6.4 million cases in 2021, with countries such as India, China, Indonesia, the Philippines, Pakistan, Nigeria and South Africa accounting for approximately 60% of the cases [2]. Pulmonary TB is caused by *Mycobacterium tuberculosis* (Mtb), a bacterium that affects the lungs but can also affect extra-pulmonary sites. Exposed individuals may immunologically clear the infection or present with active TB or latent tuberculosis infection (LTBI), which at some point in life may progress to active TB disease [3, 4].

Management of active TB disease using a long-term combination of narrow and broad-spectrum rifampicin-based antibiotics has increased the cure rate from 80% to approximately 90% and significantly reduced mortality [2]. Globally, during the COVID-19 pandemic, 1.6 million people died during or after the course of TB treatment and about 7% relapsed [2, 3]. This has been largely attributed to the interruption of TB service provision due to COVID-19, poor adherence, drug resistance and, lately, hyperglycemia [5, 6]. According to recent literature, during TB infection and the intensive phase of treatment, *M*. *tuberculosis* and the rifampicin drug alter insulin activity, distort carbohydrate metabolism, and impair glucose tolerance thus increasing blood glucose levels [7, 8]. Additionally, there is hyperglycemia due to increased hepatic glucose production and insulin resistance as a result of cytokine and hormone production disturbances [9]. This may be attributed to psychological stress associated with TB disease, anticipated long-term treatment, and social stigma [9, 10]. Continuous drug- and pathogen-induced hyperglycemia creates a non-diabetes glycemic state (intermediate hyperglycemia), increasing the risk of TB treatment failure, relapse, multidrug-resistant TB, and T2DM among the non-diabetes mellitus (DM) -TB patients [10].

Pre-diabetes and T2DM patients are increasing in many parts of the world, including sub-Saharan Africa, where TB is endemic [11]. According to International Federation of Diabetes (IDF) report published in 2021, new cases of DM are projected to rise from 567 million to 643 million cases by 2030 [10, 11]. This increase may be attributed to dietary change, sedentary lifestyle, increasing ageing generation population and partly to factors associated with infectious diseases [10–12]. Individuals presenting with normoglycemia harbour a complex community of microorganisms in the lungs that co-evolve with human cells to induce the immune system and maintain health [5, 13]. The microbiota stimulates and regulates production of mucus, antimicrobial peptides, immunoglobulins, and cytokines to recruit inflammatory cells that promote epithelial resistance and limit pathogen access to host tissues [5, 14]. Several studies, including animal model studies, have reported an association between hyperglycemia and excessive production of mucus, structural lung changes such as fibrosis and destruction or dilation of the alveoli and pulmonary inflammation and infection [5, 15]. However, information on whether TB infection, treatment and related factors predisposes an individual to T2DM and whether hyperglycemia has any effect on the lung microbiome and mucus production is scarce. The few available studies, mostly systematic reviews, report a total burden of TB-associated hyperglycemia of above 10% with 6.8% presenting with T2DM after a follow up of 3 to 6 months [11].

The intricate interplay between TB and DM and the increasing associated morbidity and mortality, especially in low- and middle-income countries, has fostered a collaborative framework between the IDF, the World Health Organization (WHO), and the International Union Against Tuberculosis and Lung Disease (IUATLD) to support and recommend bidirectional and baseline screening of DM among TB patients [2, 11, 16, 17]. In this systematic review, we aim to synthesize literature on the impact of tuberculosis-induced hyperglycemia on lung microbiota in individuals not previously diabetic and build on existing knowledge on sputum microbiota of treatment-naïve and follow up TB patients. We also propose to synthesize literature on mucin secretion in association with lung microbiome and quantify the relationship using meta-analysis. The systematic review and meta-analysis will provide baseline information to aid in screening TB patients for DM, identify gaps, and propose future research in clinical and public health.

## Methods

### Protocol design and registration

The PRISMA-P 2015 checklist was used to write this protocol [18] (S1 Checklist). The protocol has been registered with the International Prospective Register of Systematic Reviews (PROSPERO), CRD42024482248. The PRISMA 2020 checklist will be used when writing the full systematic review.

### Literature search strategy

Electronic search will be conducted on PubMed, EMBASE, MEDLINE, PROQUEST, SCOPUS, and manually on Google Scholar. We will check references of relevant articles to further identify other studies. Key search terms will be Tuberculosis, dysglycemia AND (OR) diabetes mellitus, “mucin secretion”, “mucus production”, microbiome AND (OR) microbiota. Different search strings, including Boolean operators (OR, AND), suffix truncation (*) and quotation marks (“”) for phrases will be used during the search.

### Study selection

**Fig 1:**
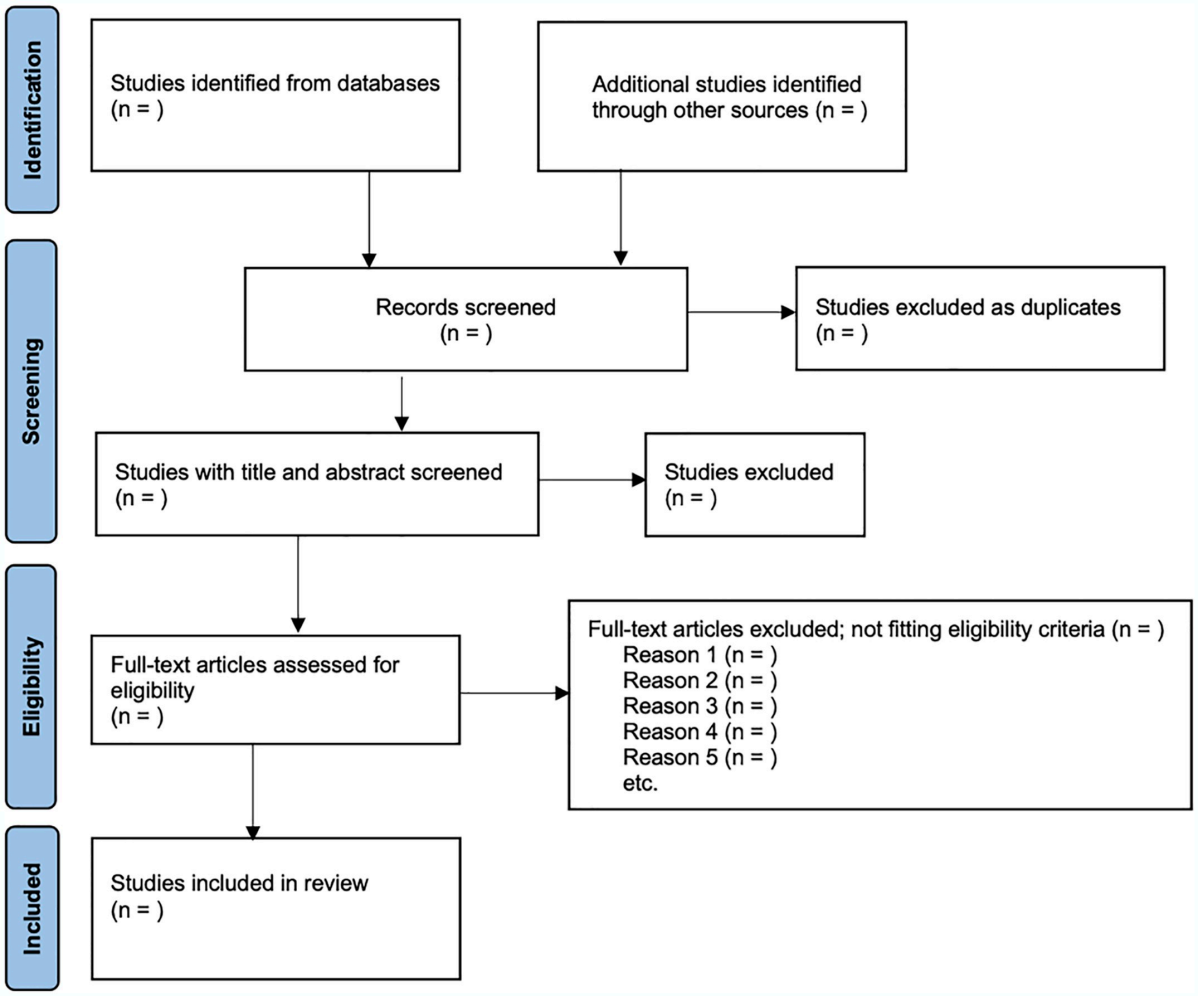
PRISMA flow diagram for study selection.

As demonstrated by the PRISMA flow chart (Fig 1), for eligibility, studies will be included if:

1. Peer-reviewed and written in English or have an English translation.
2. Prospective cohort studies with outcome of interest (microbial diversity and mucin secretion levels) measured more than once, before and during TB treatment. Cross-sectional studies, case reports, case series, and studies that have one point outcome measurement will be excluded. Retrospective studies will be excluded as the control group is recruited by convenience sampling, thus not a representative group. Additionally, in cases of missing data, articles with missing data may differ significantly from those with complete data. Underrepresentation of the population of interest and missing data will influence the outcome estimates and lead to invalid conclusions; hence this will have an impact on policy decisions and future research.
3. Involving human participants above the age of 18 years; newly diagnosed TB patients (positive chest X-ray and (or) bacteriologically confirmed sputum sample) without DM and on conventional TB treatment. Changes in mucin secretion levels and lung microbiota after initiating TB treatment will be compared to baseline (newly diagnosed treatment-naïve TB patients). We will exclude studies that recruited participants who had TB relapse and recurrence.
4. Describing the glycemic status. Hyperglycemia as the exposure of interest is defined as Fasting blood glucose >5.6mmol/L, impaired glucose tolerance– 2-hour Oral glucose tolerance test result of >7.8mmo/L and (or) HBA1C levels of 5.7%– 6.4%). Normoglycemia, the control group defined based on HBA1C levels of <5.6%; Fasting blood glucose 3.9mmol/L—5.6mmol/L and (or) 2-hour Oral glucose tolerance test result of <7.8mmol/L) [19].
5. Based on the following outcome: change in microbial diversity and mucin secretion levels compared within and between the exposed and unexposed group, before and during TB treatment period.

### Data extraction and management

Studies that meet the eligibility criteria will be selected using a multi-stage screening method. First, the titles and abstracts will be screened against the eligibility criteria; followed by screening of the full text of potentially relevant studies. The screening and selection of studies will be conducted independently by two reviewers (VMM and KN). Discrepancies, if any, will be resolved through consensus by consulting an expert in this field (EMO), as a third reviewer (EMO). Studies that will have a highly biased selection criteria based on consensus by the reviewers will be excluded. Data relevant to the objective will be extracted into MS Excel spreadsheet by VMM and confirmed by KN. We will summarise the included studies by recording the following parameters: first author, publication year, country, study design, study period, study population, mean or median age, TB diagnosis method, baseline and time of follow-up, method used to ascertain the hyperglycemia, number, and percentage of hyperglycemic and normoglycemic baseline and follow-up cases. Additional parameters will include sequencing methods, sequencing region of the 16sRNA or whole genome sequencing, microbial diversity, mucin secretion and (or) airway surface liquid and method used. For any missing information, authors will be contacted via email where possible.

### Data analysis

To assess the quality of the studies included, we will use the Modified Newcastle-Ottawa Scale (NOS) [20]. This will focus on selection—based on exposure status and representativeness (having outcome of interest); comparability—criteria used to decide on the exposed and unexposed cohort; and ascertainment of exposure. Using the quality appraisal scale, studies will be scored out of 10. Studies with a score of 7 and above will be rated as good quality, 4 to 6 as average quality, while 0 to 3 will be considered poor quality. Three reviewers (VMM, KN and EMO) will ascertain the risk of bias using Cochrane Risk of Bias Tool (CRBT) and explore any selection, performance, and reporting biases using Egger’s tool [21]. Each study included will be graded as high, low, or unclear for the risk of bias and will be considered during data synthesis. The studies graded as unclear and high risk of bias will be excluded during sensitivity analysis to determine the effect of removal on the results. The statistical analysis will be performed using R software version 4.3.1. We plan to calculate the effect size of each outcome within each study, classify the studies based on their type of effect size and pool these different effect size estimates. Meta-analysis will be conducted using random effect model for heterogeneity, after assessing the heterogeneity using I^2^ statistic (>50% will be considered high), to pool estimates on microbial diversity (phylum and genus level) in normoglycemic and hyperglycemic cases. Mean (pooled) total mucin concentration, proportion of the microbiome (phylum level), and overall estimate of microbial genera will be determined to observe the difference before and during the TB treatment period within and between normoglycemic and hyperglycaemic groups. Descriptive and meta-analysis findings will be presented using tables and forest plots.

## Discussion

This systematic review and meta-analysis, to the best of our knowledge, will provide the first narrative and quantitative information on the impact of tuberculosis-induced hyperglycemia on lung microbiota and mucus secretion in individuals not previously diabetic. The study will explore the existing scientific literature on TB-induced hyperglycemia, pulmonary microbial composition, and mucus secretion. The findings from this review, including the pooled estimates, will be disseminated in a peer-reviewed journal. It will also be shared with clinicians, scientists, and policymakers to guide and inform on the impact of persistent hyperglycemia among TB patients on host-microbe interaction, specifically bacterial proliferation in a mucus-rich environment, which may complicate TB disease management. The findings will provide baseline information on TB-induced hyperglycemia and its impact on lung microbiota and secretion of airway mucus in individuals not previously diabetic. This will attempt to demonstrate that high glucose concentration alters the composition of the lung microbiota and airway homeostasis, exerting an influence on TB disease and treatment outcomes. Additionally, this systematic review will contribute to the existing knowledge on sputum microbiota in TB disease and identify gaps for future research in clinical and public health.

### Strengths and limitations

The major strength of this study is that the selection of the studies will be done using a multi-stage screening method. The screening and selection will be performed by two independent reviewers, with a third reviewer, an expert in this field, resolving discrepancies, if any, through consensus. In addition, the wide and comprehensive search within six databases and the manual search on Google Scholar and references of relevant articles to identify other studies to include all articles of interest. The anticipated limitation is the heterogeneity in the sample size, sensitivity, and specificity of screening and microbial sequencing methods. Microbial diversity may differ within the hypervariable-target sequencing method and between whole genome sequencing, presenting a challenge in result comparison. Additionally, inclusion of English-only texts due to limitations in translation will reduce the availability of data for extraction.

## Supporting information

S1 Checklist TB-induced hyperglycemia. Preferred Reporting Items for Systematic review and Meta-Analysis Protocols 2015 checklist completed for this systematic review study protocol.
(DOCX)

## References

[1] Baker MA, Harries AD, Jeon CY, Hart JE, Kapur A, Lönnroth K, et al. The impact of diabetes on tuberculosis treatment outcomes: A systematic review. BMC Med. 2011;9(1):81. doi: 10.1186/1741-7015-9-81 21722362 PMC3155828

[2] WHO. Global Tuberculosis Report. 2023.

[3] UNAIDS. Tuberculosis and HIV. Vol. 54, Joint United Nations Programme on HIV/AIDS. 2018.

[4] Odera S, Mureithi M, Aballa A, Onyango N, Anzala O, Oyugi J. Latent tuberculosis among household contacts of pulmonary tuberculosis cases in Nairobi, Kenya. Pan Afr Med J. 2020;37(87):1–14. doi: 10.11604/pamj.2020.37.87.21102 33244350 PMC7680229

[5] Namasivayam S, Sher A, Glickman M, Wipperman M. The Microbiome and Tuberculosis: Early Evidence for Cross Talk. Am Soc Microbiol. 2018;9(5):1–11. doi: 10.1128/mBio.01420-18 30228238 PMC6143735

[6] Majigo M, Somi G, Joachim A, Manyahi J, Nondi J, Sambu V, et al. Prevalence and incidence rate of tuberculosis among HIV-infected patients enrolled in HIV care, treatment, and support program in mainland Tanzania. Trop Med Health. 2020;48(76):1–8. doi: 10.1186/s41182-020-00264-1 33579394 PMC7818072

[7] Mao F, Chen T, Zhao Y, Zhang C, Bai B, Zhao S, et al. Insulin resistance: A potential marker and risk factor for active tuberculosis? Med Hypotheses. 2011;77(1):66–8. doi: 10.1016/j.mehy.2011.03.025 21459520

[8] Philips L, Visser J, Nel D, Blaauw R. The association between tuberculosis and the development of insulin resistance in adults with pulmonary tuberculosis in the Western sub-district of the Cape Metropole region, South Africa: A combined cross-sectional, cohort study. BMC Infect Dis. 2017;17(1):1–12. 28810840 10.1186/s12879-017-2657-5 PMC5556352

[9] Menon S, Rossi R, Dusabimana A, Zdraveska N, Bhattacharyya S, Francis J. The epidemiology of tuberculosis-associated hyperglycemia in individuals newly screened for type 2 diabetes mellitus: systematic review and meta-analysis. BMC Infect Dis. 2020;20(1):937. doi: 10.1186/s12879-020-05512-7 33297969 PMC7724718

[10] Crevel R van Critchley JA. The interaction of diabetes and tuberculosis: Translating research to policy and practice. Trop Med Infect Dis. 2021;6(8):1–17. 10.3390/tropicalmed6010008 PMC7838867 33435609

[11] IDF. International Diabetes Federation: Diabetes Atlas. 2021.

[12] Chen Z, Liu Q, Song R, Zhang W, Wang T, Lian Z, et al. The association of glycemic level and prevalence of tuberculosis: a meta-analysis. BMC Endocr Disord. 2021;21(1):123. doi: 10.1186/s12902-021-00779-6 34134685 PMC8207612

[13] Lawani MB, Morris A. The respiratory microbiome of HIV-infected individuals. Expert Rev Anti Infect Ther. 2016;14(8):719–29. doi: 10.1080/14787210.2016.1206469 27348261 PMC4980914

[14] Morgan PA, Parbie PK, Ntiamoah DO, Boadu AA, Asare P, Lamptey INK, et al. Gut microbiome variation in pulmonary TB patients with diabetes or HIV comorbidities. Front Microbiomes. 2023; 2:1123064. 10.3389/frmbi.2023.1123064 PMC12993506 41853362

[15] Ye SB, Choi YS, Choi YH, Bae CH, Kim YW, Park SY, et al. Effect of high glucose on MUC5B expression in human airway epithelial cells. Clin Exp Otorhinolaryngol. 2017;10(1):77–84. doi: 10.21053/ceo.2016.00045 27384035

[16] Kateete DP, Mbabazi MM, Nakazzi F, Katabazi FA, Kigozi E, Ssengooba W, et al. Sputum microbiota profiles of treatment-naïve TB patients in Uganda before and during first-line therapy. Sci Rep. 2021;11(1):24486. 34966183 10.1038/s41598-021-04271-y PMC8716532

[17] Magee MJ, Salindri AD, Kyaw NTT, Auld SC, Haw JS, Umpierrez GE. Stress Hyperglycemia in Patients with Tuberculosis Disease: Epidemiology and Clinical Implications. Curr Diab Rep. 2018;18(9):71. doi: 10.1007/s11892-018-1036-y 30090969 PMC6309553

[18] Shamseer L, Moher D, Clarke M, Ghersi D, Liberati A, Petticrew M, et al. Preferred reporting items for systematic review and meta-analysis protocols (PRISMA-P) 2015: elaboration and explanation. BMJ. 2015 Jan 2;349: g7647–g7647. Available from: https://www.bmj.com/lookup/doi/10.1136/bmj.g7647 25555855 10.1136/bmj.g7647

[19] Liu Y, Li J, Wu Y, Zhang H, Lv Q, Zhang Y, et al. Evidence from a Systematic Review and Meta-Analysis: Classical Impaired Glucose Tolerance Should Be Divided into Subgroups of Isolated Impaired Glucose Tolerance and Impaired Glucose Tolerance Combined with Impaired Fasting Glucose, according to the Risk. Front Endocrinol. 2022;13. 10.3389/fendo.2022.835460 PMC8894674 35250886

[20] Wells GB, O’Connell D, Peterson J, Welch V, Losos M, Tugwell P. The Newcastle-Ottawa Scale (NOS) for assessing the quality of nonrandomised studies in meta-analyses 2018. http://www.ohri.ca/programs/clinical_epidemiology/oxford.asp.

[21] Higgins JP, Savović J, Page MJ, Sterne JAC. Revised Cochrane risk of bias tool for randomized trials. BMJ. 2016;(2.0).

